# Comprehensive Molecular Characterization of the Mitochondrial Genome of the Takin Lungworm *Varestrongylus eleguneniensis* (Strongylida: Protostrongylidae)

**DOI:** 10.3390/ijms232113597

**Published:** 2022-11-06

**Authors:** Yue Xie, Yijun Chen, Lidan Wang, Zhao Wang, Pengchen Zhu, Zun Hu, Xiaobin Gu, Ran He, Jing Xu, Bo Jing, Xuerong Peng, Guangyou Yang, Xuan Zhou

**Affiliations:** 1 Department of Parasitology, College of Veterinary Medicine, Sichuan Agricultural University, Chengdu 611130, China; 2 Department of Food Technology and Science, College of Food Science, Shanghai Ocean University, Shanghai 201306, China; 3 Department of Chemistry, College of Life and Basic Science, Sichuan Agricultural University, Chengdu 611130, China

**Keywords:** *Varestrongylus* lungworm, takins, mitogenome

## Abstract

The takin lungworm *Varestrongylus eleguneniensis* (Strongylida: Protostrongylidae) causes lethal bronchopneumonia and represents severe threats to captive and wild populations. However, until now there has been very limited information available concerning the molecular epidemiology and evolutionary biology of *V. eleguneniensis*. Mitochondrial genomes (mtDNAs) can provide resources for investigations in these areas and, therefore, can assist with the surveillance and control of this lungworm. Herein, the complete mtDNA of *V. eleguneniensis* was sequenced and characterized with Illumina pipeline analyses. This circular genome (13,625 bp) encoded twelve protein-coding genes (PCGs), two rRNAs, and twenty-two tRNAs, with notable levels of AT and GC skews. Comparative genomics revealed a purifying selection among PCGs, with *cox1* and *nad6* having the lowest and the highest evolutionary rate, respectively. Genome-wide phylogenies showed a close relationship between *V. eleguneniensis* and *Protostrongylus rufescens* in Strongylida. Single gene (PCGs or rRNAs)-based phylogenies indicated that *cox1* and *nad5* genes shared the same family-level topology with that inferred from genomic datasets, suggesting that both genes could be suitable genetic markers for evolutionary and phylogenetic studies of Strongylida species. This was the first mtDNA of any member of the genus *Varestrongylus,* and its comprehensive molecular characterization represents a new resource for systematic, population genetic and evolutionary biological studies of *Varestrongylus* lungworms in wildlife.

## 1. Introduction

The takin (*Budorcas taxicolor*) is considered endangered by the International Union for Conservation of Nature Criteria (IUCN) and endemic to high elevation regions ranging from the Eastern Himalayas to South-Central China [1]. The nematode *Varestrongylus eleguneniensis* (Strongylida: Protostrongylidae) is one of the commonest endoparasites found in the lung of the takin and represents a significant threat to captive and wild populations [2]. Like other lungworms, *V. eleguneniensis* adults parasitize in the alveoli and bronchus of the takin, where they mate and reproduce first-stage larvae (L1)-containing eggs. Then, the L1 larvae hatch, travel up the bronchus to the oral cavity, enter the intestinal tract and are finally shed into feces. The terrestrial snails ingest L1s and become their intermediate hosts, where the L1s further develop into infective third-stage larvae (L3s). Takins, as the definitive host, infected *V. eleguneniensis* by digestion of L3-containing snails when grazing [3]. Clinically, *V. eleguneniensis* infection causes pulmonary complications including lung collapse and bronchopneumonia, and in severe cases host death, especially when co-infections with dictyocaulidosis [4].

Current diagnosis of *V. eleguneniensis* infections typically relies on morphological examination [5,6,7], and key morphological features of *V. eleguneniensis* adults include the fine cuticle markings with one blunt and rounded tip and four papillae around the buccal foramen. However, the morphology is often unrecognized, even by experienced parasitologists, and mistaken identification, particularly of the eggs and larvae, is not uncommon [8]. Additionally, the diagnosis becomes more challenging when identification and differentiation of eggs or larvae are carried out within an environment cross-contaminated by other lungworm eggs and larvae, including morphologically similar *Dictyocaulus* spp. [9,10]. Therefore, obtaining a more efficient and reliable method to detect *V. eleguneniensis* eggs or larvae has become crucial for clinical diagnosis, epidemiological survey, and laboratory examination, and achieving this goal is foreseeable only through the application of molecular methodologies.

Recent methods using ribosomal or mitochondrial DNA have proven valuable complementary tools to overcome this problem and are widely utilized for species identification of many Strongylida species, including lungworms [11,12]. For example, Verocai et al. studied the prevalence of lungworms from muskoxen and caribous and molecularly identified *V. eleguneniensis* as a widespread, multi-host species of wild North American ungulates based on sequencing and genetically analyzing the ribosomal internal transcribed spacer 2 (*ITS-2*) [13]. Considering that the mitochondrial DNA (mtDNA) could be more suitable than the ribosomal sequences for species identification owing to its maternal inheritance, high mutation, and lack of recombination [14], Li and colleagues determined the mitochondrial *cox1* gene of Tibetan pig lungworms and verified that all lungworms were *Metastrongylus pudendotectus* [15]. Furthermore, Jabbar et al. applied the mitochondrial *cox1*, *cytb*, and *rrnS* genes-based multiplex PCR to monitor the prevalence of *Dictyoculus* lungworms and demonstrated the presence of *Dictyoculus cervi* infection in the Polish moose population [16]. However, compared to single mitochondrial gene loci, the complete mtDNA data can provide the genome-level genetic information and, therefore, would yield novel insights into evolutionary and phylogenetic-based analyses of lungworms.

Until now, there has been no information available on the mtDNAs of any *Varestrongylus* species. In this study, we sequenced, *de novo* assembled and characterized the complete mtDNA of *V. eleguneniensis*, the first member of the genus *Varestrongylus*, using Illumina technology. Then, the structure and organization of *V. eleguneniensis* mtDNA were compared with those of other Strongylida species to explore the conservation and variability of the whole genome at the nucleotide- and amino acid-levels. Following comparative genomics, the phylogenetic relationships of *V. eleguneniensis* in the family Protostrongylidae and of the family Protostrongylidae within the order Strongylida were also studied by reconstruction of phylogenetic trees (maximum parsimony (MP), maximum likelihood (ML), and Bayesian inference (BI)) using either single or concatenated datasets of twelve PCGs and two rRNAs.

## 2. Results and Discussion

### 2.1. Genome Feature of the Takin Lungworm

**Figure 1:**
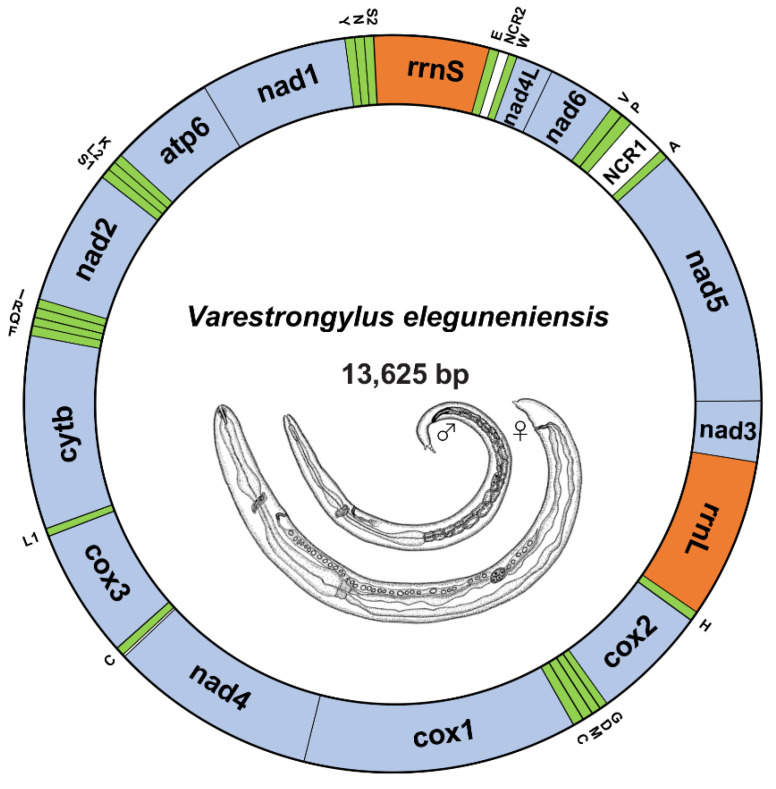
The circular map of *V. eleguneniensis* mtDNA. Genes are represented by different colored blocks. PCGs are denoted in blue, rRNAs in orange, tRNAs in green and non-coding regions in white. Gene abbreviations: *atp6*, ATP synthase subunits 6; *cox1-3*, cytochrome oxidase subunits 1-3; *cytb*, cytochrome b; *nad1-6*/*nad4L*, NADH dehydrogenase subunits 1-6/4 L; *rrnL*, large rRNA subunit; *rrnS*, small rRNA subunits; NCR1, AT-rich region; NCR2, the non-coding region. The tRNAs are indicated with their one-letter corresponding amino acids. Line drawings of *V. eleguneniensis* is adapted with permission from Ref. [13]. 2014, Guilherme G. Verocai.

The circular mtDNA of *V. eleguneniensis* was 13,625 bp in length, comparable to other sequenced Strongylida species (ranging from 13,310 to 15,221 bp) [17,18,19,20]. The entire genome encoded 36 genes, including 12 PCGs (*atp6*, *cox1-3*, *cytb*, *nad1-6*, and *nad4L*), two mitochondrial rRNA genes (small (*rrnS*) and large (*rrnL*) subunits) and 22 tRNA genes (Figure 1). All these genes were present on the same strand and transcribed in the same direction (5′ to 3′). The gene order of the mtDNA of *V. eleguneniensis* followed the gene arrangement (GA3) [21,22,23], similar to that of the other Strongylida members. Moreover, there were two non-coding regions, namely NCR1 and NCR2, presented in this circular genome. NCR1 was 192 bp in length and located between the *nad5* and *nad6* genes, flanking at the 5′-end by the *tRNA-Ala* gene and at the 3′-end by the *tRNA-Pro*. Because of its high AT content (81.3%), this region was also known as AT-region. NCR2 was 57 bp in length and located between *nad4L* and *rrnS*, flanking at the 5′-end by the *tRNA-Trp* gene and at the 3′-end by the *tRNA-Glu*. In addition, five overlapping regions, ranging from 1 bp to 21bp, were located between *cytb* and *tRNA-Leu* (UAG) (1 bp), *cox2* and *tRNA-His* (2 bp), *tRNA-His* and *rrnL* (1 bp), *tRNA-Glu* and *rrnS* (21 bp), and *tRNA-Ser* (UGA) and *tRNA-Asn* (1 bp), respectively. 140 nucleotides were observed to be scattered in 11 intergenic spacers, ranging from 1 to 36 bp in length.

### 2.2. Nucleotide Content and Codon Usage

**Figure 2:**
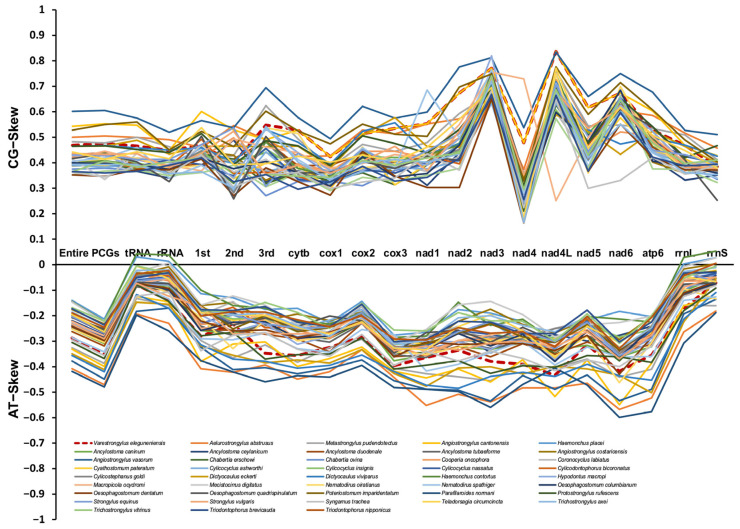
The GC and AT skews for mtDNAs of 21 different regions (entire mtDNA, 12PCGs, rRNAs, tRNAs, the 1st site in codon, the 2nd site in codon, the 3rd site in codon, *rrnL*, *rrnS*, and each single PCG) within the 43 Strongylida mtDNAs. Different species are indicated by different colored lines graph.

The nucleotide content of *V. eleguneniensis* mtDNA was 27.0% A, 48.8% T, 6.4% C, and 17.8% G, respectively, with a total of AT content of 75.9% (Table S1). This significant AT bias was similar to other Strongylida members and was within the range of AT contents described for other species in the same order (69.1 to 79.2%) [24,25]. And tRNAs and rRNAs also showed a strong AT bias with AT content of 79.17% and 78.16%, respectively. Generally, the AT and GC skews on the complementary strands of each mtDNA could be used to measure the compositional asymmetry [26]. For *V. eleguneniensis* mtDNA, the AT and GC skews were −0.287 and 0.471, respectively, and their comparisons with other 43 Strongylida species were shown in Figure 2. It was obvious that the nucleotide compositions of mtDNAs of these species consistently exhibited an extreme skew away from A and C in favor of T and G, with −0.48 to −0.145 of AT skew and 0.348 to 0.608 of GC skew. Specifically, the respective AT and GC skews were −0.198~0.043 and 0.368~0.577 in the rRNAs, −0.259~0.039 and 0.328~0.522 in the tRNAs, and −0.480~−0.004 and 0.337~0.608 in the concatenated 12 PCGs. Notably, among the PCGs, the TG bias was also significant at the first, second, and third positions of codons with AT and GC skews being −0.408~−0.099 and 0.363~0.603, −0.421~−0.122 and 0.260~0.548, and −0.459~−0.148 and 0.273~0.696, respectively.

**Figure 3:**
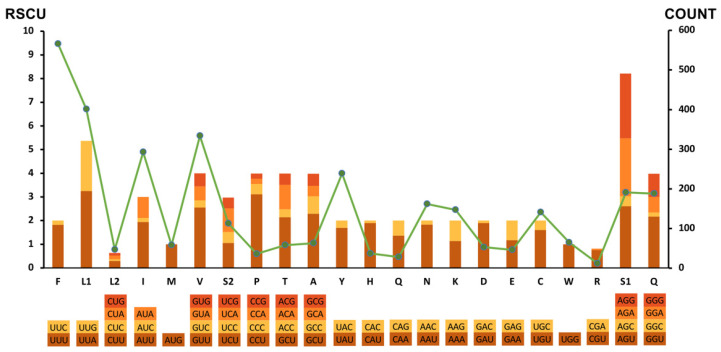
Relative synonymous codon usage (RSCU) and codon numbers in *V. eleguneniensis* mtDNA. Codon families are provided on the X-axis, and the RSCU of each codon is represented by different color bars (left axis scale). The codon numbers are indicated by the green line graph (right axis scale).

Interestingly, the genome-wide AT bias was also reflected in the relative synonymous codon usage (RSCU) and codon usage patterns of PCGs. As shown in Figure 3, among 12 PCGs, the most frequently used codon was UUA (RSCU = 3.25; N = 243), followed by AGU (RSCU = 2.61; N = 99), GAU (RSCU = 2.29; N = 50), and GUU (RSCU = 2.55, N = 213). Correspondingly, the most frequently used amino acids were Phe (UUC & UUU; N = 566), Leu (UUG & UUA; N = 401), Val (GUG & GUA & GUC & GUU; N = 334) and Ile (AUA & AUC & AUU; N = 293). Further, a similar nucleotide bias was also observed in the choices of start and stop codons. Six of twelve PCGs were inferred to start with codon TTR (TTG was used for *cytb*, *cox3*, *nad1*, *nad4*, and *nad6*, and TTA was used for *nad3*) and the other six PCGs were initiated by the codon ATW as start codon (ATT was used for *cox2*, *nad5*, *nad4L*, and *atp6*, ATA was used for *cox1* and *nad2*). In addition, six PCGs were terminated by an incomplete stop codon, and the incomplete stop codons were used for *cox3*, *nad5*, *nad1*, and *nad4L* (T-), for *cox1* and *atp6* (TA-) (Table 1). The incomplete stop codons may be converted into complete ones by post-transcriptional polyadenylation during mRNA maturation [27,28].

### 2.3. tRNAs and rRNAs

**Figure 4:**
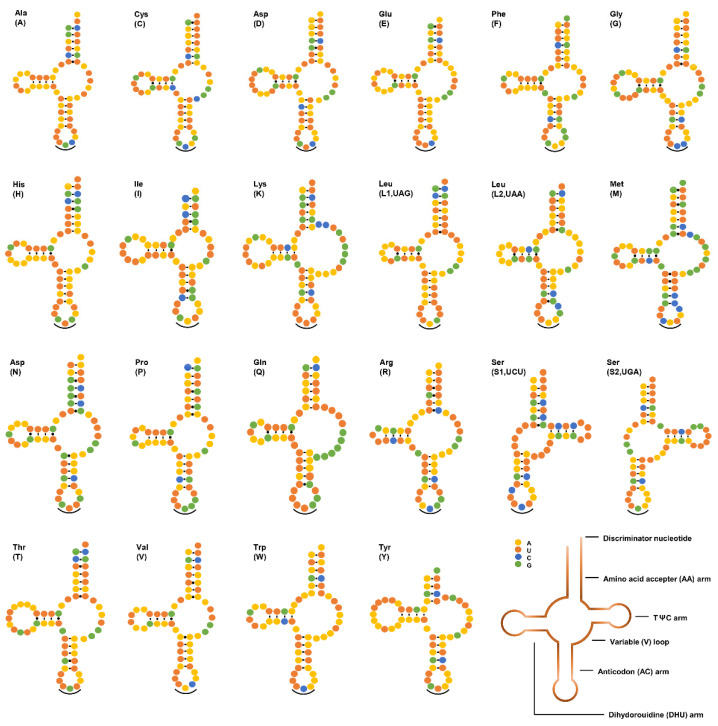
Secondary structures of 22 tRNAs in *V. eleguneniensis* mtDNA. All tRNAs are differed with their corresponding amino acid abbreviations. Each base is represented by a different colored circle, yellow for adenine (A), orange for uracil (U), green for guanine (G), and blue for cytosine (C). Normal base pairings are illustrated by lines (-), whereas non-standard base pairs are illustrated by dots (·). Anticodons are marked by black curved lines (‿).

Twenty-two tRNAs ranged from 52 to 64 bp in length, with a total length of 1263 bp in the mtDNA of *V. eleguneniensis*. Similar to other chromadorean nematodes sequenced so far [19,20,29], except for two *tRNA-Ser*, twenty tRNAs had a typical secondary structure with 4-bp DHU arms and 3~11-bp DHU loops, and its variable TψC arm and loop were replaced by a 6~12-bp TV loop (Figure 4). For these two *tRNA-Ser*, both were equipped with 4-bp TψC arms and 4~7-bp loops instead of the DHU arm and loop. Within two rRNAs, the *rrnL* gene was present between *tRNA-His* and *nad3*, and the *rrnS* was placed between *tRNA-Glu* and *tRNA-Ser* (UGA). The lengths of *rrnL* and *rrnS* were 995 bp and 722 bp, which were within the length ranges of other rRNAs published in Strongylida species with 946–983 bp for *rrnL* and 684–724 bp for *rrnS*, respectively.

### 2.4. Comparison with Other Strongylida Genomes

**Figure 5:**
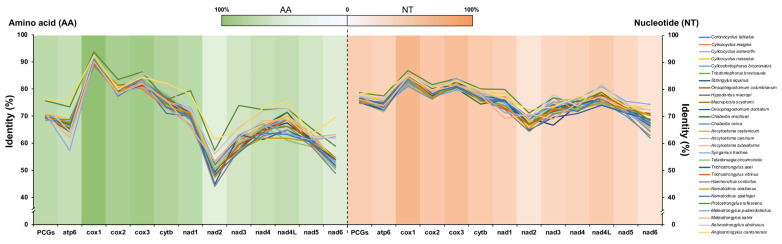
Identity comparison of PCGs for 28 related Strongylida species with *V. eleguneniensis*. Different species are indicated by different colored broken lines. The heat maps in green and orange represent the average values of sequence identities of amino acid and nucleotide datasets, respectively.

Pairwise comparisons of the 12 PCGs of mtDNAs between *V. eleguneniensis* and other Strongylida species were shown in Figure 5 and Table S2. It was clear that the lengths of 12 PCGs in *V. eleguneniensis* were similar to those of most other Strongylida nematodes. Specifically, the *atp6* gene among the PCGs was the most conserved with 600 bp in length followed by *nad3* (336 bp) and *nad4* (1230 bp). In contrast, the lengths of the *cox1* and *nad1* genes were the most variable, with 1572~1617 bp and 825~924 bp, respectively. Further sequence identity analyses showed that 74.7~78.7% nucleotide and 68.5~76.1% amino acid identities of 12 PCGs between *V. eleguneniensis* and other Strongylida species. It appeared that *V. eleguneniensis* shared relatively higher levels of the nucleotide and amino acid identities (in decreasing order) with *Protostrongylus rufescens* (78.7% and 75.6%)/*Angiostrongylus cantonensis* (78.3% and 76.1%), *Aelurostrongylus abstrusus* (77.5% and 73.5%), *Ancylostoma tubaeforme* (77.5% and 71.1%), and *Ancylostoma caninum* (77.4% and 71.0%). For each PCG, *cox1* and *cox3* were the most conserved genes because their high nucleotide (81.1~86.9% for *cox1* and 79.6~84.1% for *cox3*) and amino acid (88.4~93.7% for *cox1* and 80.0~86.3% for *cox3*) identities, whereas *nad2* and *nad6* were the most variable genes because their low nucleotide (64.4~72.4% for *nad2* and 62.0~74.4% for *nad6*) and amino acid (44.1~61.3% for *nad2* and 48.9~69.5% for *nad6*) identities.

**Figure 6:**
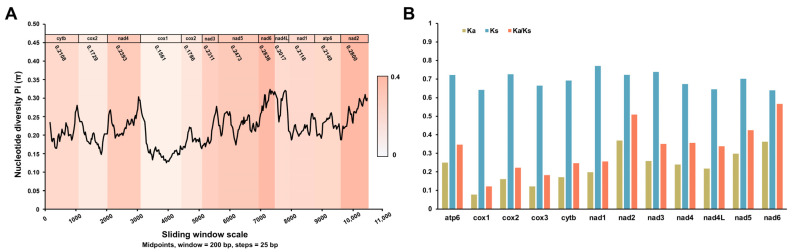
Evolutionary diversity analyses of the mtDNA among 40 Strongylida species including *V. eleguneniensis*. (**A**), Sliding window analyses of 12 PCGs. The black line shows the value of nucleotide diversity (Pi) in a sliding window analysis (200-bp sliding window with 25-bp step size); the Pi values of each gene are shown under the gene name and reflected by the heat maps on the background; (**B**), The evolutionary rates of 12 PCGs in Strongylida mtDNAs. The Ka, Ks, and Ka/Ks are calculated for each PCG.

To further explore the diversities within and between PCGs, the sliding window analysis was employed using the nucleotide alignments of *V. eleguneniensis* and other Strongylida species. As shown in Figure 6A, the values of the nucleotide diversity Pi of PCGs ranged from 0.123 to 0.321 among Strongylida species included here. Combined with the calculation of the number of variable positions per unit length of the gene, the *nad6* was determined as the most variable gene (Pi = 0.2838), followed by *nad2* (Pi = 0.2800), *nad5* (Pi = 0.2473), and *nad3* (Pi = 0.2311). However, the *cox1* was determined as the most gene conserved with 0.1561 of Pi. This finding was consistent with the result of the identity analysis, which further demonstrates the accuracy of data from this study.

In parallel, Ka, Ks, and the Ka/Ks ratios were also calculated for all PCGs among the aforementioned Strongylida nematodes in order to estimate their evolutionary rates [30]. As shown in Figure 6B, the *nad6* among the 12 PCGs showed the maximum ratio of Ka/Ks (0.566), followed by *nad2* (0.509), *nad5* (0.425), *nad4* (0.356), and *atp6* (0.375). Nevertheless, these ratios all were less than 1. In general, the ratio of Ka/Ks is often regarded as a measure of selective pressure of PCGs and indicates neutral mutation when Ka/Ks = 1, negative or purifying selection when Ka/Ks < 1 and positive or diversifying selection when Ka/Ks > 1 [31,32]. Therefore, it was reasonable that these PCGs in Strongylida were all subjected to a purifying selection during their evolutions.

### 2.5. Phylogenetic Analysis

**Figure 7:**
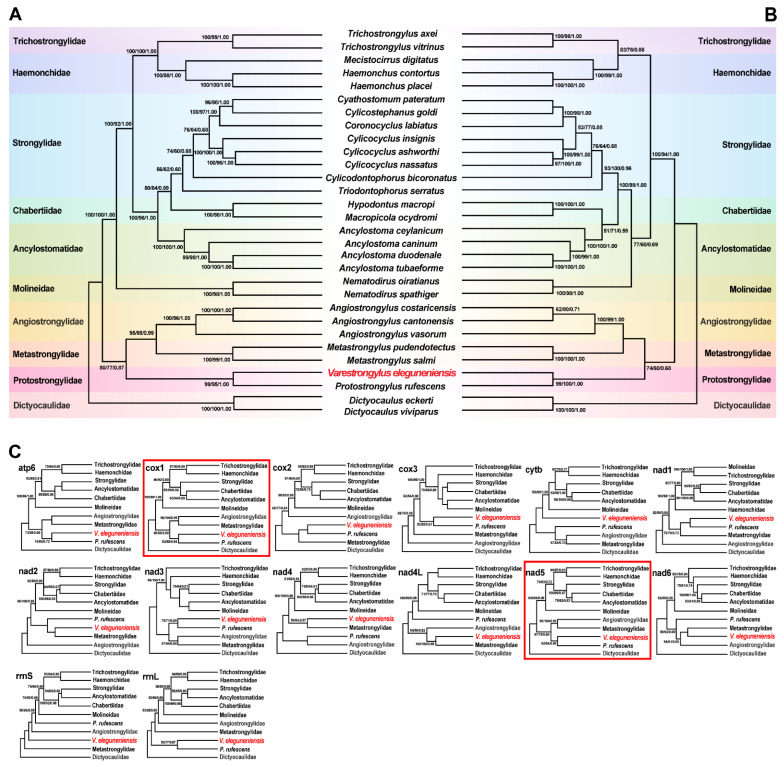
Phylogenies were inferred using three methods (MP, ML, and BI) based on mtDNA datasets containing 12 PCGs and two rRNAs. *Dictyocaulus eckerti* and *Dictyocaulus viviparus* are used as the outgroups. Different colored regions represent different families. (**A**), Phylogenetic tree based on the concatenated nucleotide sequences of the 12 PCGs; (**B**), Phylogenetic tree based on the concatenated amino acid sequences of the 12 PCGs; (**C**), Phylogenetic trees based on nucleotide sequences of 14 individual genes (12 PCGs and two rRNAs). The phylogenetic trees consistent with the trees in (**A**,**B**) are marked with red line boxes. The species sequenced in this study is shown in red fonts. The numbers along the branches indicate bootstrap values/posterior probabilities resulting from different analyses in the order MP/ML/BI.

The available mtDNA of *V. eleguneniensis* provided us an opportunity to study the evolutionary relationships of *V. eleguneniensis* in the family Protostrongylidae and of the family Protostrongylidae within the order Strongylida. As shown in Figure 7A,B, it was clear that three identical trees (MP/ML/BI) inferred from either the concatenated nucleotide or amino acid datasets consistently revealed that compared to other Strongylida species, *V. eleguneniensis* shared the closest relationship with *P. rufescens*, with high statistical support (all values ≥ 95 or = 1.00), consistent with previously proposed molecular phylogeny based on the *ITS-2* [13]. Further, *V. eleguneniensis* and *P. rufescens* under the family Protostrongylidae clustered with species of the families Angiostrongylidae and Metastrongylidae, and together formed one branch and exhibited a paraphyletic relationship with species of the families Trichostrongylidae, Haemonchidae, Strongylidae, Chabertiidae, Ancylostomatidae, and Molineidae, supporting recent mtDNA-based phylogenetic conclusion [33,34,35,36].

In addition, single gene-based phylogenetic analyses were used to scoop out the optimal genetic marker candidates among the PCGs and rRNAs for phylogeny and species identification. As shown in Figure 7C, although most of PCGs and rRNAs showed different phylogenetic topologies, the positions of the family Protostrongylidae, Metastrongylidae, and Angiostrongylidae were steady in the *cox1*-, *cytb*-, *nad1*-, *nad3*-, *nad5*-, and *nad6*-based phylogenetic analyses. Notably, the *cox1* and *nad5* genes shared the same phylogenetic topology as that of the genome-based phylogenetic analysis, suggesting that both genes might be the most appropriate genetic markers and, therefore, could be used instead of mtDNA for evolutionary and phylogenetic studies of this parasite and other related species in Strongylida. Certainly, the marker validity of the *cox1* and *nad5* remains further validated when more additional Strongylida mtDNAs become available, especially those from the genus *Varestrongylus*, although the *cox1* has been widely used as a DNA barcode for species identification and differentiation in parasitic nematodes [37,38].

## 3. Materials and Methods

### 3.1. Parasite Sampling

**Figure 8:**
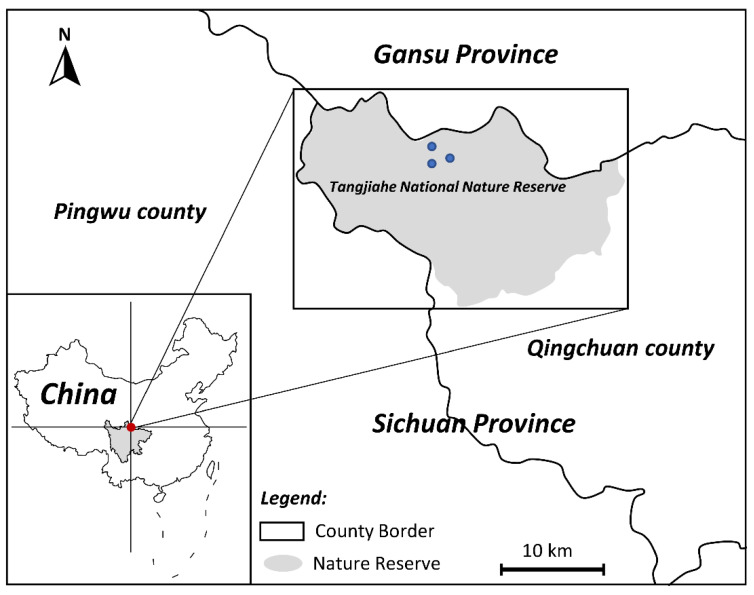
Sampling plots of takin lungworms collected from Tangjiahe National Nature Reserve, China. The red and blue dots on the map are the location of the Tangjiahe National Nature Reserve and the sampling points, respectively.

From September to October 2020, there were three dead wild *B. taxicolor* adults found in Tangjiahe National Nature Reserve, Sichuan, China (Figure 8); one of them was then brought to the Veterinary Medical Teaching Hospital, Sichuan Agricultural University (Ya’an, China), for postmortem examination. These takins died with the same clinical manifestations, including dyspnea, coughing and wheezing. After a standard necropsy under the Scientific Procedures Premises License for the College of Veterinary Medicine, Sichuan Agricultural University (SYXK 2014-187), diseased lungs were dissected, and about 56 nematode specimens were collected from the respiratory tracts of lungs. Through washing in physiological saline, all worms were preliminarily identified as *V. eleguneniensis* according to morphological keys [3]. Then, two specimens were further chosen for molecular identification by PCR amplifying the *ITS-2* region, followed by sequence comparison with the previously reported corresponding sequence for *V. eleguneniensis* (GenBank accession number: JX115006) [13]. A result of 99.8% sequence identity of the *ITS-2* between both worms and *V. eleguneniensis* confirmed their species identity.

### 3.2. V. eleguneniensis mtDNA Sequencing and Annotation

To decode the mtDNA of *V. eleguneniensis*, the total genomic DNA was extracted from pooled lungworm specimens (n = 20) using the Genomic DNA Extraction Kit ver. 3.0 (TaKaRa Biotech, Dalian, China). After DNA yield and integrity evaluation, a 0.25-μg aliquot of genomic DNA was fragmented, end-paired, and ligated to adaptor. The ligated fragments were isolated on agarose gels and purified by PCR amplification to produce the sequencing library. A 350-bp paired-end (PE) library was constructed and sequenced using the Illumina HiSeq TM 4000 platform (Illumina, San Diego, CA, USA). The raw reads were quality-trimmed by the following criteria: filtered reads with adapters, filtered reads with N bases >15 bp, and filtered reads with low-quality bases (≤3). In total, 2.5 Gb of clean data (250 bp each, paired-end reads) were obtained and assembled using MITObim v1.9 software [39] with default parameters, followed by manual inspection for searching the overlapping regions to circularize the complete mtDNA. This mtDNA was further verified by PCR amplifications using nine overlapping segments (ranging in length from 1.42 kp to 2.33 kb), which were designed on the basis of the alignments of the relatively conserved regions of the available Strongylida mtDNAs. The corresponding primer sequences are shown in Table S3. PCR reactions were achieved using a 50 μL reaction volume containing 3 μL genomic DNA (≥15 ng), 25 μL 2 × HiFi TransTaq PCR SuperMix, 1 μL sense primer (10 pmol), 1 μL anti-sense primer (10 pmol), and 20 μL nuclease-free water. PCR conditions were 5 min denaturation at 95 °C, followed by 30 cycles of 45 s at 95 °C, 40 s at 45~55 °C, and 1~3 min at 68 °C according to the Tm value and the product length, with a final extension at 68 °C for 10 min. After agarose gel detections, all target amplicons were sequenced either directly or following sub-cloning into the pMD19-T vector (TaKaRa, Biotech, Dalian, China). Each amplicon was sequenced three times to ensure maximum accuracy. This consensus circular genome was drawn with MacVector ver. 9.5 and annotated using MITOS and online BLAST tools available on the NCBI website [39]. The boundaries of PCGs and rRNA genes were verified by alignment with homologous genes of other related nematodes using DNAMAN ver. 8.0 (Lynnon Biosoft, Vaudreuil-Dorion, QC, Canada). In addition to MITOS, open Reading Frame (ORF) Finder and Primer software were also used to define the amino acid sequences of PCGs and to find the start and stop codons using the invertebrate mitochondrial genetic code [40]. tRNA genes were identified using ARWEN and tRNAscan-SE [41,42], and their secondary structures were predicted and manually plotted using the software Adobe Illustrator CS5 (www.adobe.com/products/illustrator.html (accessed on 17 June 2022)). The complete sequence of *V. eleguneniensis* mtDNA was deposited in GenBank under accession number: OP464906.

### 3.3. Sequence Analysis

Nucleotide content and codon usage of *V. eleguneniensis* mtDNA were measured using Geneious and PhyloSuite [43,44]. The nucleotide skewness of *V. eleguneniensis* and their comparisons with 42 other nematodes in the order Strongylida (Table 2) were performed using the formulas [26,34,35,36,45,46,47,48,49,50,51,52,53,54,55,56,57]: AT skew = (A − T)/(A + T) and GC skew = (G − C)/(G + C). Sequence lengths and nucleotide/amino acid identities of 12 PCGs among Strongylida species were determined using DNAstar [58]. Based on the multi-alignment of 12 PCGs for 43 Strongylida mtDNAs (including *V. eleguneniensis*), the sliding window analysis was used to compute the nucleotide diversity Pi (π) using 200-bp window and 25-bp steps in DnaSP ver.5.10 [59]. The Pi value was plotted against the midpoint position of each window. Additionally, the evolutionary rate and the ratio of the nonsynonymous substitutions (Ka) and synonymous substitutions (Ks) of each PCG were also calculated using KaKs_Calculator [60].

### 3.4. Phylogenetic Analysis

To determine the classification positions of *V. eleguneniensis* in the family Protostrongylidae and of the family Protostrongylidae within the order Strongylida, the mtDNAs of twenty-nine selected Strongylida species were retrieved from GenBank, and their concatenated amino acid sequences of twelve PCGs and the nucleotide sequence of each PCG/rRNA were separately aligned using Clustal X ver. 2.0 for phylogenetic analyses. *Dictyocaulus eckerti* (GenBank accession no. NC_019809) and *Dictyocaulus viviparus* (GenBank accession no. NC_019810) were treated as the outgroups and included in each analysis. Phylogenetic analyses were performed with MP, ML, and BI. For the MP analysis, either the concatenated 12 PCGs dataset or multi-alignments of the nucleotide sequence for each PCG or rRNA were analyzed using equally weighted parsimony in PAUP* [61] and heuristic searches with a tree-bisection-reconnection (TBR) branch-swapping. 1000 replicates of Wagner trees were selected and five trees per replication were saved, followed by the harvest of the optimal topology using the Kishino–Hasegawa method. Bootstrap resampling with 1000 replications was calculated for each nodal support. For ML analyses, the optimal evolutionary model was selected with the “Auto” option on W-IQ-TREE web server (http://iqtree.cibiv.univie.ac.at (accessed on 20 June 2022)) and “TIM+F+I+G4” model was chosen for both matrices according to Bayesian Information Criterion (BIC). ML trees were constructed using an ultrafast bootstrap approximation approach with 100,000 replications. Within the BI trees, the optimal evolutionary model CAT+GTR+G was selected for mtDNA datasets using ModelFinder [62]. The BI computations were achieved using MrBayes 3.2.7 [63], with four independent Markov chains, running for 100,000 (concatenated dataset of 12 PCGs) and 10,000,000 (single PCG or rRNA dataset) metropolises coupled with Monte Carlo generations, sampling a tree every 100 and 10,000 generations. When the average standard deviation (SD) of the split frequencies was reduced to below 0.01, the first 25% trees were discarded as “burn-in” and the remaining were used to compute Bayesian posterior probabilities. The evolutionary distance was estimated using the MrBayes order (aamodelpr = mixed) with default parameters. A consensus tree was obtained and visualized using TreeviewX [64].

## 4. Conclusions

In this study, *V. eleguneniensis* mtDNA represented the first genome sequenced in the members of the genus *Varestrongylus*, and its comparison with the other Strongylida species revealed that *cox1* and *nad6* were the most and least conserved genes, respectively, across the order. Genome-wide phylogenies refined the phylogenetic relationship of *V. eleguneniensis* in the family Protostrongylidae of Strongylida and showed a close relationship between *V. eleguneniensis* and *P. rufescens*. Further single gene-based phylogenies suggested that the *cox1* and *nad5* genes could be suitable genetic markers for phylogeny and species identification in Strongylida because their topologies are the same as that of the whole genome. The results should contribute to a better understanding of systematics and evolutionary biology of *Varestrongylus* lungworms and, therefore, can assist in their prevention and control by providing resources of molecular markers for molecular diagnostic, epidemiology and population genetic studies.

## Figures and Tables

**Table 1:** Structures and organizations of *V. eleguneniensis* mtDNA.

Gene	Location	Length	Start Codon	Stop Codon	Anticodon
*cytb*	1–1110	1110	TTG	TAA	
*tRNA-Leu* (UAG)	1110–1167	58			UAG
*cox3*	1168–1933	766	TTG	T	
*tRNA-Thr*	1934–1991	58			UGU
*nad4*	1996–3225	1230	TTG	TAG	
*cox1*	3250–4865	1616	ATA	TA	
*tRNA-Cys*	4866–4921	56			GCA
*tRNA-Met*	4922–4980	59			CAU
*tRNA-Asp*	4981–5040	60			GUC
*tRNA-Gly*	5041–5099	59			UCC
*cox2*	5100–5792	693	ATT	TAG	
*tRNA-His*	5791–5846	56			GUG
*rrnL*	5846–6800	955			
*nad3*	6801–7136	336	TTA	TAA	
*nad5*	7143–8724	1582	ATT	T	
*tRNA-Ala*	8725–8779	55			UGC
*tRNA-Pro*	8972–9025	54			UGG
*tRNA-Val*	9027–9090	64			UAC
*nad6*	9094–9516	423	TTG	TAA	
*nad4L*	9517–9748	232	ATT	T	
*tRNA-Trp*	9749–9805	57			UCA
*tRNA-Glu*	9863–9920	58			UUC
*rrnS*	9900–10,621	722			
*tRNA-Ser* (UGA)	10,622–10,678	57			UGA
*tRNA-Asn*	10,678–10,740	63			GUU
*tRNA-Tyr*	10,749–10,804	56			GUA
*nad1*	10,805–11,687	884	TTG	T	
*atp6*	11,691–12,289	599	ATT	TA	
*tRNA-Lys*	12,301–12,364	64			UUU
*tRNA-Leu* (UAA)	12,401–12,455	55			UAA
*tRNA-Ser* (UCU)	12,456–12,507	52			UCU
*nad2*	12,508–13,359	852	ATA	TAA	
*tRNA-Ile*	13,386–13,439	54			GAU
*tRNA-Arg*	13,440–13,492	53			ACG
*tRNA-Gln*	13,493–13,549	57			UUG
*tRNA-Phe*	13,568–13,625	58			GAA

**Table 2:** Summary mtDNA information included in this study.

Family	Species	Host	Country	GenBank Nos.	References
Ancylostomatidae	*Ancylostoma caninum*	Dog	South Africa	NC_012309	[45]
Ancylostomatidae	*Ancylostoma ceylanicum*	Hamster	Solomon Islands	AP017674	Unpublished
Ancylostomatidae	*Ancylostoma duodenale*	Human	China	NC_003415	[46]
Ancylostomatidae	*Ancylostoma tubaeforme*	Cat	China	NC_034289	[47]
Angiostrongylidae	*Aelurostrongylus abstrusus*	Cat	Australia	NC_019571	[33]
Angiostrongylidae	*Angiostrongylus cantonensis*	Rat	China	AP017672	Unpublished
Angiostrongylidae	*Angiostrongylus costaricensis*	Rat	Japan	AP017675	Unpublished
Angiostrongylidae	*Angiostrongylus vasorum*	Dog	China	NC_018602	[48]
Chabertiidae	*Chabertia erschowi*	Yak	China	NC_023782	[49]
Chabertiidae	*Chabertia ovina*	Sheep	Australia	GQ888721	[34]
Chabertiidae	*Hypodontus macropi*	Kangaroo	Australia	KF361317	[50]
Chabertiidae	*Macropicola ocydromi*	Wallaby	Australia	KF361320	[50]
Chabertiidae	*Oesophagostomum asperum*	Goat	China	KC715826	[51]
Chabertiidae	*Oesophagostomum columbianum*	Sheep	China	KC715827	[51]
Chabertiidae	*Oesophagostomum quadrispinulatum*	Pig	China	FM161883	[52]
Chabertiidae	*Oesophagostomum dentatum*	Pig	China	FM161882	[52]
Cooperiidae	*Cooperia oncophora*	Cow	Netherlands	AY265417	[53]
Dictyocaulidae	*Dictyocaulus eckerti*	Cattle	Sweden	NC_019809	[54]
Dictyocaulidae	*Dictyocaulus viviparus*	Cattle	Sweden	NC_019810	[54]
Filaroididae	*Parafilaroides normani*	Fur seal	Australia	KJ801815	[55]
Haemonchidae	*Haemonchus placei*	Sheep	Japan	AP017687	Unpublished
Haemonchidae	*Mecistocirrus digitatus*	Sheep	China	NC_013848	[34]
Haemonchidae	*Teladorsagia circumcincta*	Sheep	Australia	GQ888720	[34]
Haemonchidae	*Haemonchus contortus*	Sheep	Australia	EU346694	[35]
Metastrongylidae	*Metastrongylus pudendotectus*	Pig	Estonia	NC_013813	[34]
Metastrongylidae	*Metastrongylus salmi*	Pig	Estonia	GQ888715	[34]
Molineidae	*Nematodirus oiratianus*	Goat	China	KF573750	[56]
Molineidae	*Nematodirus spathiger*	Sheep	China	KF573749	[56]
Protostrongylidae	*Protostrongylus rufescens*	Sheep	Australia	KF481953	[36]
Strongylidae	*Coronocyclus labiatus*	Horse	China	MH551242	Unpublished
Strongylidae	*Cyathostomum pateratum*	Horse	China	NC_038070	Unpublished
Strongylidae	*Cylicocyclus ashworthi*	Horse	China	NC_046711	[57]
Strongylidae	*Cylicocyclus insignis*	Horse	Australia	NC_013808	[34]
Strongylidae	*Cylicocyclus nassatus*	Horse	Australia	NC_032299	Unpublished
Strongylidae	*Cylicodontophorus bicoronatus*	Horse	China	MH551241	Unpublished
Strongylidae	*Poteriostomum imparidentatum*	Horse	China	NC_035005	Unpublished
Strongylidae	*Strongylus equinus*	Horse	China	KM605251	Unpublished
Strongylidae	*Strongylus vulgaris*	Horse	Australia	GQ888717	[34]
Strongylidae	*Triodontophorus brevicauda*	Horse	China	NC_026729	Unpublished
Strongylidae	*Triodontophorus nipponicus*	Horse	China	NC_031517	Unpublished
Strongylidae	*Triodontophorus serratus*	Horse	China	NC_031516	Unpublished
Strongylidea	*Cylicostephanus goldi*	Horse	Japan	AP017681	Unpublished
Syngamidae	*Syngamus trachea*	Magpie	Australia	NC_013821	[34]
Trichostrongylidae	*Trichostrongylus axei*	Sheep	Australia	NC_013824	[34]
Trichostrongylidae	*Trichostrongylus vitrinus*	Sheep	Australia	NC_013807	[34]

## Data Availability

Molecular data have been deposited to GenBank with the following accession number: OP464906.

## Supplementary Materials

The following supporting information can be downloaded at: https://www.mdpi.com/article/10.3390/ijms232113597/s1.
