# Supplementary material for: Comprehensive Molecular Characterization of the Mitochondrial Genome of the Takin Lungworm Varestrongylus eleguneniensis (Strongylida: Protostrongylidae)
Source: Int J Mol Sci. 2022 Nov 6;23(21):13597. doi: 10.3390/ijms232113597 (PMC9658269; doi:10.3390/ijms232113597)
Supplement: Supplementary file 1 [file ijms-23-13597-s001.zip › ijms-2014645-supplementary.pdf]

International Journal of Molecular Sciences

MDPI

Table S1. Nucleotide contents of the mtDNAs of the forty-five Strongylida nematodes.

Species	GenBank Nos.	Whole Genome				
		A%	T%	G%	C%	A+T%
Ancylostoma caninum	NC_012309	29.0	48.5	16.1	6.5	77.5
Ancylostoma ceylanicum	AP017674	30.6	47.9	15.0	6.4	78.5
Ancylostoma duodenale	NC_003415	28.3	48.4	16.7	6.6	76.7
Ancylostoma tubaeforme	NC_034289	29.2	48.5	15.6	6.7	77.7
Aelurostrongylus abstrusus	NC_019571	21.3	50.6	21.1	7.0	71.9
Angiostrongylus cantonensis	AP017672	24.5	48.7	20.7	6.1	73.2
Angiostrongylus costaricensis	AP017675	26.7	48.2	18.8	6.3	74.9
Angiostrongylus vasorum	NC_018602	21.5	47.6	24.7	6.1	69.1
Chabertia erschowi	NC_023782	28.3	46.0	17.8	7.8	74.3
Chabertia ovina	GQ888721	29.6	46.7	16.3	7.4	76.3
Hypodontus macropi	KF361317	29.3	46.9	16.1	6.5	76.2
Macropicola ocydromi	KF361320	29.4	46.4	17.4	7.2	75.8
Oesophagostomum asperum	KC715826	30.2	47.5	15.4	6.9	77.7
Oesophagostomum columbianum	KC715827	29.6	48.1	15.0	7.2	77.7
Oesophagostomum quadrispinulatum	FM161883	30.7	46.8	15.2	7.3	77.5
Oesophagostomum dentatum	FM161882	21.0	51.2	17.0	7.2	72.2
Cooperia oncophora	AY265417	30.1	47.3	16.1	6.5	77.4
Dictyocaulus eckerti	NC_019809	24.9	51.6	17.4	6.3	76.5
Dictyocaulus viviparus	NC_019810	24.6	51.8	17.3	6.3	76.4
Parafilaroides normani	KJ801815	21.0	51.2	21.2	6.5	72.2
Haemonchus placei	AP017687	34.1	45.2	14.5	6.2	79.3
Mecistocirrus digitatus	NC_013848	34.0	45.7	14.4	5.9	79.7
Teladorsagia circumcincta	GQ888720	31.0	46.1	15.6	7.3	77.1
Haemonchus contortus	EU346694	33.4	44.7	15.4	6.5	78.1
Metastrongylus pudendotectus	NC_013813	26.1	51.4	16.7	5.8	77.5
Metastrongylus salmi	GQ888715	23.7	50.3	14.4	5.9	74.0
Nematodirus oiratianus	KF573750	28.5	47.7	16.8	7.1	76.2
Nematodirus spathiger	KF573749	26.8	48.3	17.6	7.3	75.1
Protostrongylus rufescens	KF481953	25.9	48.6	18.7	6.8	74.5
Coronocyclus labiatus	MH551242	30.5	45.4	16.9	7.2	75.9
Cyathostomum pateratum	NC_038070	30.5	45.2	16.9	7.4	75.7
Cylicocyclus ashworthi	NC_046711	30.2	45.2	17.1	7.5	75.4
Cylicocyclus insignis	NC_013808	30.6	46.0	16.6	6.8	76.6
Cylicocyclus nassatus	NC_032299	29.7	45.1	17.7	7.6	74.8
Cylicodontophorus bicoronatus	MH551241	29.9	45.3	17.4	7.4	75.2
Poteriostomum imparidentatum	NC_035005	29.8	44.9	18.0	7.4	74.7
Strongylus equinus	KM605251	31.1	47.0	15.7	6.2	78.1
Strongylus vulgaris	GQ888717	29.3	47.2	17.8	7.0	76.5
Triodontophorus brevicauda	NC_026729	31.9	45.2	16.4	7.6	77.1

Triodontophorus nipponicus	NC_031517	30.8	45.2	16.4	7.6	76.0
Triodontophorus serratus	NC_031516	31.8	45.4	15.9	6.9	77.2
Cylicostephanus goldi	AP017681	30.5	45.6	16.8	7.1	76.1
Syngamus trachea	NC_013821	26.0	47.8	18.8	7.4	73.8
Trichostrongylus axei	NC_013824	31.1	45.3	17.0	6.6	76.4
Trichostrongylus vitrinus	NC_013807	32.8	46.1	14.7	6.3	78.9

Table S2. Gene lengths in the mtDNAs of the forty-five reported Strongylida nematodes.

Species	GenBank No.	Length(bp)														
		Entire mtDNA	*atp6*	*cox1*	*cox2*	*cox3*	*cytb*	*nad1*	*nad2*	*nad3*	*nad4*	*nad4L*	*nad5*	*nad6*	*rrnL*	*rrnS*
Ancylostoma caninum	NC_012309	13,717	600	1578	696	776	1113	870	846	336	1230	234	1582	432	963	694
Ancylostoma ceylanicum	AP017674	13,655	600	1587	696	766	1113	872	846	336	1230	234	1588	435	960	683
Ancylostoma duodenale	NC_003415	13,721	600	1577	696	766	1112	873	846	336	1230	234	1579	435	960	697
Ancylostoma tubaeforme	NC_034289	13,730	600	1578	696	766	1112	873	846	336	1230	234	1576	435	958	697
Aelurostrongylus abstrusus	NC_019571	13,913	600	1578	693	772	1104	867	855	339	1230	237	1602	426	961	697
Angiostrongylus cantonensis	AP017672	13,506	600	1617	693	766	1110	876	849	336	1230	232	1582	426	957	724
Angiostrongylus costaricensis	AP017675	13,645	600	1587	693	766	1110	873	848	334	1230	232	1585	429	968	698
Angiostrongylus vasorum	NC_018602	13,422	600	1575	699	771	1110	879	846	336	1224	233	1567	429	962	697
Chabertia erschowi	NC_023782	13,705	600	1578	696	766	1113	873	846	339	1230	234	1582	435	970	696
Chabertia ovina	GQ888721	13,682	597	1578	696	771	1113	873	840	336	1233	234	1582	431	962	700
Hypodontus macropi	KF361317	13,655	600	1572	696	766	1115	855	849	336	1200	234	1661	435	960	701
Macropicola ocydromi	KF361320	13,659	600	1572	696	766	1113	855	834	339	1206	234	1561	429	960	701
Oesophagostomum asperum	KC715826	13,672	600	1578	696	766	1113	873	846	336	1230	234	1582	435	964	697
Oesophagostomum columbianum	KC715827	13,561	599	1577	696	766	1111	873	845	336	1230	234	1582	435	961	694
Oesophagostomum quadrispinulatum	FM161883	13,681	600	1578	696	766	1113	873	846	336	1230	234	1582	435	961	699
Oesophagostomum dentatum	FM161882	13,752	600	1578	696	766	1113	873	846	336	1230	234	1582	435	966	700
Cooperia oncophora	AY265417	13,636	600	1581	699	766	1111	871	835	334	1230	234	1582	438	949	696
Dictyocaulus eckerti	NC_019809	13,300	585	1575	690	795	1081	825	825	315	1170	232	1567	417	960	701
Dictyocaulus viviparus	NC_019810	13,310	600	1581	687	774	1078	883	817	336	1227	235	1570	438	962	697
Parafilaroides normani	KJ801815	13,414	594	1572	696	766	1103	830	859	348	1191	258	1511	435	962	697
Haemonchus placei	AP017687	14,251	600	1582	693	766	1113	846	870	342	1230	232	1582	446	946	715
Mecistocirrus digitatus	NC_013848	15,221	600	1576	699	772	1111	873	846	336	1230	234	1582	432	959	700
Teladorsagia circumcincta	GQ888720	14,066	600	1578	696	769	1111	873	840	336	1212	232	1579	438	959	700
Haemonchus contortus	EU346694	14,055	600	1582	693	769	1113	873	846	336	1220	232	1582	441	952	703

Metastrongylus pudendotectus	NC_013813	13,793	603	1575	696	771	1116	924	850	342	1221	240	1586	437	959	700
Metastrongylus salmi	GQ888715	13,778	597	1575	696	771	1113	891	853	342	1230	234	1584	438	959	700
Nematodirus oiratianus	KF573750	13,765	598	1576	694	766	1113	867	838	327	1230	229	1582	438	962	696
Nematodirus spathiger	KF573749	13,591	600	1576	696	766	1112	867	839	327	1230	229	1582	438	958	695
Protostrongylus rufescens	KF481953	13,619	600	1572	693	775	1104	876	848	336	1230	234	1575	426	960	684
Coronocyclus labiatus	MH551242	13,827	600	1578	696	766	1110	873	846	336	1230	234	1584	435	979	701
Cyathostomum pateratum	NC_038070	13,822	600	1575	696	766	1113	873	846	336	1230	234	1584	435	976	698
Cylicocyclus ashworthi	NC_046711	13,876	600	1578	696	766	1113	873	846	336	1230	234	1584	435	978	711
Cylicocyclus insignis	NC_013808	13,828	600	1578	696	769	1113	873	846	336	1230	234	1584	435	959	700
Cylicocyclus nassatus	NC_032299	13,846	600	1578	696	766	1113	873	846	336	1230	234	1584	435	974	699
Cylicodontophorus bicoronatus	MH551241	13,753	600	1578	696	766	1113	873	846	336	1230	234	1584	435	982	702
Poteriostomum imparidentatum	NC_035005	13,817	600	1578	696	766	1113	873	846	336	1227	234	1584	435	983	709
Strongylus equinus	KM605251	14,545	600	1578	696	766	1113	879	846	336	1230	234	1599	435	959	708
Strongylus vulgaris	GQ888717	14,301	603	1578	696	766	1116	876	846	336	1230	234	1584	435	959	700
Triodontophorus brevicauda	NC_026729	14,305	600	1578	696	766	1113	873	846	336	1230	234	1584	435	975	703
Triodontophorus nipponicus	NC_031517	13,701	600	1578	696	766	1113	873	846	336	1230	234	1584	435	976	696
Triodontophorus serratus	NC_031516	13,794	600	1578	696	766	1113	873	846	336	1230	234	1584	435	961	701
Cylicostephanus goldi	AP017681	13,827	600	1578	696	766	1113	873	846	336	1230	234	1593	435	972	699
Syngamus trachea	NC_013821	14,647	600	1578	696	775	1116	873	849	324	1230	234	1582	435	959	700
Trichostrongylus axei	NC_013824	13,653	600	1578	693	769	1113	864	846	336	1227	234	1582	435	959	700
Trichostrongylus vitrinus	NC_013807	13,800	597	1578	693	766	1113	864	846	336	1227	234	1582	435	959	700

Table S3. List of the nine primer pairs for PCR amplification and their positions in *V. eleguneniensis* mtDNA.

Primer Name	Location in *V. eleguneniensis* mtDNA	Primer Sequence (5' to 3')
VE-1	13,435-13,454	Forward: GGTGAAATGTTAAATAGAGT
	1232-1255	Reverse: TTACTAAAGAACTAGTTAAACTTA
VE-2	918-941	Forward: GTCTGTGTTGGCTAAATTAAATAA
	2312-2335	Reverse: GCGCACCAAAACCTAAGATTATAA
VE-3	2070-2095	Forward: AGGTATGTCAATATTAAATAATATTT
	3462-3485	Reverse: ATAATAGAATTATATAACTGACCA
VE-4	3112-3133	Forward: GTTTGTTCGTGTATGGGAAAAA
	5221-5244	Reverse: CCCCTCTTATCAACAACAAAAATC
VE-5	4836-4857	Forward: TTATTTTAGTAGAAGTAGTTTA
	6999-7024	Reverse: CCCACAAATATAACAATTTCTAAATC
VE-6	6748-6771	Forward: ATTAGTACGAAAGGAAAGTTAATT
	8399-8422	Reverse: CTAGGTATTCCAATTATATTAACA
VE-7	8274-8293	Forward: CGTCTGTGAAAAAGTTTTTT
	10,580-10,604	Reverse: CCCCTAAATCTACTTTACTACAACT
VE-8	10,406-10,428	Forward: AGATATATATTTAGTTTATAAAT
	11,904-11,928	Reverse: TTCCACAAACACAAAAAGAATAAGA
VE-9	11,763-11,785	Forward: GGGATTTTGAGTGTGTTTTTTGA
	13,519-13,538	Reverse: CCCCCCTTTACACCAAAAAT
